# An Exploratory Retrospective Study on the Association of Radiotherapy with the Risk of Immune-Related Adverse Events in Esophageal and Esophagogastric Junction Cancer Patients Receiving Immunotherapy

**DOI:** 10.3390/cancers17243992

**Published:** 2025-12-15

**Authors:** Nobukazu Hokamura, Takeo Fukagawa, Ryoji Fukushima, Takashi Kiyokawa, Masahiro Horikawa, Yuichi Igarashi, Hironori Midorikawa, Shinya Kaneshiro, Kenshiro Shiraishi

**Affiliations:** 1 Department of Surgery, Teikyo University School of Medicine, Tokyo 173-0003, Japan; tfukagaw@yahoo.co.jp (T.F.); ryojif@med.teikyo-u.ac.jp (R.F.); takashikiyokawa@med.teikyo-u.ac.jp (T.K.); hori0131@hotmail.co.jp (M.H.); igarashi.yuichi.vf@teikyo-u.ac.jp (Y.I.); kxbxg369@yahoo.co.jp (H.M.); kinjo-d@outlook.jp (S.K.); 2 Department of Radiology, Teikyo University School of Medicine, Tokyo 173-0003, Japan; shiraishiken@med.teikyo-u.ac.jp

**Keywords:** esophageal cancer, radiotherapy, immune checkpoint inhibitor, irAEs, lymphocyte depletion, mediastinal irradiation

## Abstract

Background: Radiotherapy (RT) combined with immune checkpoint inhibitors (ICIs) has shown therapeutic benefits, and the potential for enhanced immune activation has raised concerns about increased immune-related adverse events (irAEs). The immunological implications of mediastinal RT combined with ICI therapy remain unclear. Methods: We conducted an exploratory retrospective review of 58 patients with esophageal oresophagogastric junction cancer who received ICIs between 2021 and 2024. Patients were categorized into RT (+) and RT (-) groups based on whether they underwent mediastinal RT. The incidence and severity of irAEs were compared using chi-square testing. Subgroup analyses included treatment sequence (RT before vs. after ICI), interval between RT and ICI (<90 vs. ≥90 days), and ICI regimen (nivolumab [N], pembrolizumab [P], or nivolumab plus ipilimumab [NI]). Results: irAEs occurred in 28.6% of RT (+) and 39.1% of RT (-) (*p* = 0.42). Severe irAEs were uncommon in both groups. Treatment sequence and RT-ICI interval did not significantly influence irAE incidence. irAEs were more frequent in the NI group (85.7%) than in N (22.9%) or P (31.2%) (*p* = 0.01). Mediastinal RT itself did not increase irAE risk. Conclusions: Although RT combined with ICIs has been hypothesized to elevate irAEs through enhanced immune activation, mediastinal RT did not increase irAEs in this cohort. However, given the exploratory and small patient cohort, these findings suggest, with caution, that mediastinal irradiation may attenuate systemic immune activation through lymphocyte depletion, potentially balancing ICI-induced immune responses.

## Simple Summary

Radiotherapy and immune checkpoint inhibitors (ICIs) are often used in the treatment of esophageal cancer. However, whether radiotherapy influences the risk of immune-related adverse events (irAEs) during ICI therapy has not been well clarified. This question is particularly relevant in esophageal cancer because the radiation field often includes the mediastinum, which contains a large pool of circulating lymphocytes and lymphatic drainage pathways. In this study, we evaluated patients with esophageal and esophagogastric junction cancer who received ICIs, with or without mediastinal radiotherapy. We initially hypothesized that combining radiotherapy and ICIs might increase both the frequency and severity of irAEs, as has been suggested in other cancer types. However, mediastinal radiotherapy did not increase irAE incidence or severity; nevertheless, these results should be interpreted cautiously due to the exploratory, retrospective, and single-institutional nature of the study and its limited patient numbers.

## 1. Introduction

In 2011, an antibody targeting cytotoxic T lymphocyte-associated protein 4 (CTLA-4) on T cells opened up a new window for tumor treatment [1]. Since this breakthrough, several immune checkpoint inhibitors (ICIs), including programmed cell death 1 (PD-1) and its ligand programmed cell death ligand 1 (PD-L1), have been widely used for various tumors due to their sustained anti-tumor response and significant efficacy. However, some cancer cells exhibit primary resistance to ICIs, whereas others develop resistance through upregulation of PD-L1 during ICI treatment [2].

Radiotherapy has played an important role in esophageal and esophagogastric junction (EGJ) cancers, serving both palliative and curative purposes. Its anti-tumor efficacy is achieved not only by directly destroying tumor cells through DNA damage, but also by activating the immune response against cancer cells. Radiotherapy enhances tumor-specific immunity through several mechanisms, including the induction of neoantigens, upregulation of the major histocompatibility complex (MHC), and increased PD-L1 expression. The combination of radiotherapy and ICIs has been extensively studied and implemented, particularly in the treatment of non-small-cell lung cancer [3,4,5]. Though this combination can enhance anti-tumor efficacy, it may also lead to more severe irAEs.

In the treatment of esophageal and EGJ cancers, the mediastinum is typically included in the radiation field. Within the mediastinum, the thymus, located in the upper region, and the highly developed lymphatic network play crucial roles in the immune system. Irradiation to this area may negatively impact immune activity. Several studies have reported a poor prognosis in patients whose total T lymphocyte count decreased after chemoradiotherapy [6,7]. Radiotherapy for patients with esophageal and EGJ cancers, particularly when administered concomitantly with chemotherapy, can have both positive and negative effects on immune responses. Consequently, whether the combination of radiotherapy and ICIs increases the incidence and the severity of irAEs in these patients remains unclear.

To date, no studies have investigated irAEs following radiotherapy and ICI treatment in patients with esophageal and EGJ cancers. To address this gap, a retrospective review of patients who underwent radiotherapy and ICI treatment at our hospital was performed.

## 2. Materials and Methods

### 2.1. Patients

Patients with esophageal and EGJ cancers who underwent ICI treatment at our hospital from 2021 onward were retrospectively reviewed. The following information was collected from medical records: age, sex, tumor location, tumor histology, tumor classification according to the UICC-TNM classification 8th edition, type of treatment, recurrence patterns, radiation dose and field (for patients who received radiotherapy), and details of ICI treatment. Regarding irAEs, the side-effect profile was collected from medical records and graded with the use of CTCAE version 5.0. In patients with multiple irAEs, the adverse event with the highest grade was considered the representative event for that case.

### 2.2. ICI Regimens

In Japan, nivolumab was the first ICI approved for patients with unresectable, advanced, and recurrent esophageal and EGJ cancers in 2020. Between 2021 and 2022, combination therapies, including nivolumab with cisplatin and 5-fluorouracil, nivolumab with oxaliplatin and S1, pembrolizumab with cisplatin and 5-fluorouracil, and nivolumab with ipilimumab, were also approved for these patients. Currently, these regimens are administered for primary unresectable diseases, recurrent disease after surgery, and residual or recurrent disease after radiotherapy (including chemoradiotherapy). Based on these treatments, patients were categorized into three groups: the nivolumab group (monotherapy or in combination with chemotherapy, group “N”), the pembrolizumab group (monotherapy or in combination with chemotherapy, group “P”), and the dual ICI chemotherapy (nivolumab and ipilimumab, group “NI”). ICI combination therapies have become standard for patients with unresectable or advanced disease. For those with recurrent or residual disease, the choice among monotherapy, combination with chemotherapy, or dual ICI therapy was determined based on tumor characteristics and the patient’s overall condition.

### 2.3. Radiotherapy

Radiotherapy at our hospital is delivered using intensity-modulated radiation therapy (IMRT) with a Versa HD linear accelerator (Elekta, Stockholm, Sweden) and volumetric-modulated arc therapy (VMAT) with a Harmony linear accelerator (Elekta). For curative cases, low-dose hyperfractionated ionizing radiation (1.8 Gy × 28) is administered, with a total dose of 50 Gy or higher delivered in combination with chemotherapy. For palliative treatment of swollen lymph nodes, a standard dose of 30 Gy (3 Gy × 10) is used, and this regimen is likewise delivered using either IMRT or VMAT. The antitumor effect is dependent on radiation dose, and the incidence and severity of irAEs may also be influenced by both the radiation dose and the irradiation field. In the present series, the minimum radiation dose was 24 Gy. Based on the clinical consensus of our radiation oncologists, we determined this dose to be the minimum threshold for potential immunological effect, as a dose lower 20 Gy was considered This dose was not considered negligible. Therefore, all patients who received any radiation dose (≥24 Gy) were classified as RT (+), whereas those who did not receive radiotherapy were classified as RT (-). Patients who received radiation to anatomical regions outside the mediastinum, including the abdominal or cervical lymph nodes, were excluded from the analysis.

### 2.4. Definition of Immune-Related Adverse Events

Because this was a retrospective study, irAEs were primarily identified from the electronic medical records based on the clinical judgment of the treating physician. Each adverse event was evaluated by the responsible physician, and events considered compatible with an immune-related mechanism were documented. The severity of all documented adverse events was graded using the Common Terminology Criteria for Adverse Events (CTCAE) version 5.0.

To ensure a standardized determination, we focused on typical organ-specific irAEs-such as thyroid dysfunction, adrenal insufficiency, pneumonitis, dermatitis, cytokine release-related reactions, and gastrointestinal toxicities-following the American Society of Clinical Oncology (ASCO) and European Society for Medical Oncology (ESMO) guidelines for the management of toxicities [8,9]. Theses specific events can generally be distinguished from chemotherapy- or radiation-induced toxicities. To minimize misclassification, we employed strict exclusion criteria for attribution: Events with uncertain attribution were not classified as irAEs. Additionally, hematologic, hepatic, and renal abnormalities were excluded from the irAEs analysis because they are usually attributed to concomitant chemotherapy.

### 2.5. Statistics

To evaluate the effects of radiotherapy on the incidence and severity of irAEs in patients receiving ICI treatment, the RT (+) and RT (-) groups were compared. In addition, the following were analyzed in the RT (+) group to clarify their impact: (1) the sequence of radiotherapy and ICI treatment; and (2) the interval between radiotherapy and ICI treatment. Based on a previous report, the interval between radiotherapy and ICI treatment was categorized as short interval if it was less than 90 days and long interval if it was 90 days or more [10]. Finally, the three ICI groups were compared to address their effect on the incidence and severity of irAEs. Statistical analyses were performed using SPSS Statistics version 23 (IBM Corp., Armonk, NY, USA). The chi-square test was used for statistical analysis. This study was approved by the Ethics Committee of Teikyo University School of Medicine. Written informed consent was obtained from all subjects prior to their participation in this study.

## 3. Results

### 3.1. Patients

Fifty-eight patients were reviewed. Thirty-five patients underwent radiotherapy to the mediastinum. There were no significant differences in average age and the distribution of clinical stages between the RT (+) and RT (-) groups. All patients in the RT (+) group had squamous cell carcinoma of the thoracic esophagus. Twenty-eight patients initially received ICI treatment to control unresectable and/or advanced disease, and half of them later underwent radiotherapy. Ten patients received ICI treatment for recurrence after surgery, including one who had undergone chemoradiotherapy before surgery and received ICI treatment after postoperative recurrence. Twenty patients underwent ICI treatment for recurrence after chemoradiotherapy or radiotherapy (Table 1).

### 3.2. ICI Treatment

There was no significant difference in the distribution of ICI groups in the RT (+) and RT (-) groups (*p* = 0.763). At our hospital, pembrolizumab with cisplatin and fluorouracil was the standard regimen for patients with unresectable disease, followed by chemoradiotherapy after ICI treatment.

### 3.3. irAEs

A summary of non-immune-related adverse events in the RT (+) and RT (-) groups is shown in Table 2. Details of irAEs in each group are also shown in Table 2. The most common irAE was adrenal dysfunction, followed by thyroid dysfunction and pneumonitis. Severe cytokine release syndrome (CRS) developed in both groups, with one patient in the RT (-) group dying from CRS. A bleeding gastric ulcer occurred in one patient of the RT (-) group, and a causal relationship with ICI treatment could not be ruled out. The incidence of all irAEs was 28.6% in the RT (+) and 39.1% in the RT (-) groups, and the incidence of Grade ≥ 3 irAEs was 11.4% in the RT (+) group and 13.0% in the RT (-) group; neither difference was statistically significant (Table 2).

### 3.4. Analysis Within the RT (+) Group

The effect of the sequence of radiotherapy and ICI treatment was analyzed in the RT (+) group, and the results are shown in Table 3. The incidence of all irAEs was 23.8% in patients who underwent radiotherapy first and 35.7% in those who underwent ICI treatment first, with no significant difference between the groups (*p* = 0.474) (Table 3).

The effect of the interval between radiotherapy and ICI treatment was also analyzed. The incidence of all irAEs was 27.8% in patients whose interval was less than 90 days and 23.5% in those with an interval longer than 90 days; there was no significant difference (*p* = 0.71) (Table 4).

### 3.5. Type of ICI Treatment

The above analysis suggested that radiotherapy had no effect on the incidence or severity of irAEs in patients receiving ICI treatment. Therefore, the impact of the type of ICI treatment was analyzed in all patients. The number of patients in the N, P, and NI groups was 35, 16, and 7, respectively. The incidence of all irAEs in each group was 22.9%, 31.2%, and 85.7%, respectively. The incidence in patients receiving dual ICI chemotherapy was significantly higher than in the other two groups (*p* = 0.01) (Table 5).

## 4. Discussion

In this exploratory, retrospective, single-institutional study, we investigated whether mediastinal RT is associated with an increased risk or severity of irAEs. In Japan, thoracic esophageal squamous cell carcinoma accounts for nearly 90% of esophageal cancers, whereas adenocarcinoma of the esophagus or EGJ represents less than 10% of cases and is generally not treated with mediastinal radiotherapy. As a result, patients who receive mediastinal irradiation are almost exclusively those with thoracic squamous cell carcinoma. This background difference in histology and tumor location between the RT (+) and RT (-) groups reflects the clinical patterns in Japan and should be considered when interpreting our findings.

The ICI era has brought a new paradigm to cancer therapy whereby the immune system is being harnessed to cure cancer. These agents have dramatically improved disease outcomes in many patients with cancer, since they can lead to responses as sustained as to last, in some cases, for the entire patient’s lifetime [11]. ICIs exert their anti-tumor effects by inhibiting T-cell suppressive signals and subsequently activating T-cell–mediated cytotoxicity. However, during ICI treatment, resistance to ICIs can emerge due to excessive expression of PD-L1 antibodies induced by an immune response to cancer, leading to a decrease in CD8+ T lymphocytes, or due to tumor-induced changes in the tumor microenvironment (TME) [12]. However, ICIs may overactivate the immune system, leading to an increase in the levels of inflammatory factors in the microenvironment and inducing a systemic inflammatory response [13]. The delivery of these immunotherapeutic agents has been known to cause fatal adverse events [14,15].

Radiotherapy plays a vital role in both curative and palliative treatment of most types of cancer. It has been estimated that more than 50% of patients with cancer will receive radiotherapy [16]. Radiotherapy exerts an anti-tumor effect by direct DNA damage. During radiotherapy, ionizing radiation activates damage repair cascades in normal tissues, including DNA damage response and a perpetual cytokine cascade, including oxidative stress, vascular damage, and inflammatory responses. Radiotherapy also activates the immune system by inducing neoantigen expression, promoting PD-L1 expression, increasing MHC, and altering the TME [4]. These effects result in tumor regression outside of the radiation field (the so-called “abscopal effect”) [17]. However, radiotherapy can upgrade PD-L1 expression, which results in downregulation of immune activity and acquired resistance to radiotherapy [18].

Considering the effects of radiotherapy on the immune system, combining radiotherapy with ICIs is expected to have a synergistic effect. Upregulation of tumoral PD-L1 may be overcome by PD-L1 blockade. Consolidation treatment with an anti-PD-L1 antibody, durvalumab, following concurrent chemoradiotherapy has become a new standard of care for locally advanced non-small cell lung cancer [19]. Radiotherapy can make cold tumors hot by expressing neoantigens, promoting PD-L1 expression, increasing MHC, and altering the TME. Therefore, radiotherapy enhances the efficacy of ICIs. When radiotherapy is combined with ICI treatment, abscopal responses have been documented at increasing rates in patients exhibiting primary or acquired resistance to ICIs before receiving radiotherapy [4,20].

In esophageal cancer, radiotherapy, when combined with chemotherapy, is an important modality with curative potential. The Radiation Therapy Oncology Group (RTOG) 85-01 trial indicated that chemoradiotherapy should be the standard of care for unresectable, locally advanced esophageal cancer [21]. The ESMO guidelines and NCCN guidelines recommend neoadjuvant chemoradiotherapy or definitive chemoradiotherapy as the standard treatment for advanced esophageal squamous cell carcinoma [22,23]. However, the mediastinum and lymph nodes are inevitably involved in the radiation field in patients with esophageal cancer. The thymus, in the upper mediastinum, has an important role in our immune response through maturation and differentiation of T lymphocytes. Irradiation to the thymus can affect our immune system. The mediastinum contains large vessels (the aorta and pulmonary vessels); therefore, many lymphocytes are irradiated. Lymphocytes are highly sensitive to ionizing radiation, and radiotherapy has been shown to suppress the immune system.

In esophageal cancer, several clinical trials are ongoing to investigate the combination of radiotherapy and ICI treatment [24]. As discussed above, the treatment effect will be improved by synergistic effects, leading to an increase in the incidence and severity of irAEs. However, if the negative effects of radiation on lymphocytes outweigh the synergistic benefits of combination therapy, the incidence and severity of irAEs may remain unchanged or even decrease. It remains unclear whether the incidence and severity of irAEs will worsen due to irradiation of critical organs.

The present results did not show any significant impact of radiotherapy on the incidence and severity of irAEs in patients who underwent ICI treatment for esophageal and EGJ cancers. Neither the sequence of radiotherapy and ICIs nor the interval between radiotherapy and ICIs affected the incidence or severity of irAEs. However, dual ICI regimens had a stronger effect. One reason for this negative result may be the radiation technique. Low-dose hyperfractionated ionizing radiation is a standard method of chemoradiotherapy for patients with esophageal cancer. However, this method can suppress anti-tumor immunity by reducing peripheral and tumor-infiltrating effector immune subsets, increasing immunosuppressive cell subsets, and restraining PD-1/PD-L1 expression in the TME [25]. Furthermore, in patients with esophageal cancer, a wide area of the mediastinum including lymph nodes was irradiated, and many lymphocytes were affected. Consequently, immune responses were restrained, and the incidence and severity of irAEs did not worsen.

Previous studies investigating the combination of (chemo)radiotherapy and ICI treatment for patients with esophageal cancer suggested no increase in the rate of grade 3–5 adverse events compared with (chemo)radiotherapy alone [26,27]. However, the prospective PACIFIC trial [18] showed that the incidence of irAEs of any grade was 24.2% in the immunotherapy group and 8.1% in the placebo group, and the rate of patients who underwent treatments for irAEs, including systemic glucocorticoids, was 14.3% in the immunotherapy group and 5.6% in the placebo group. Although *p* values were not reported, the incidence of irAEs needing steroid treatment seemed higher in patients who underwent the combined therapy. Ansher and his colleagues concluded that there was no meaningful increase in serious irAEs in patients receiving an ICI and radiotherapy through their pooled analysis of data from 68 prospective trials [26]. However, they suggested that patients who had ICI treatment within 90 days following radiotherapy had slightly numerically higher rates of fatigue, endocrinopathies, and pneumonitis. Radiotherapy for patients with esophageal and esophagogastric junction cancers has several unique aspects in terms of the radiation field and delivery method. The combination of radiotherapy and ICIs for these patients has been further explored, and more effective combination therapies will be applied. Therefore, a prospective clinical study focusing on patients with esophageal and esophagogastric junction cancers should be conducted to determine the effects on the incidence and severity of irAEs.

## 5. Limitations

The present report was based on data from a single institution with a limited number of patients and used a retrospective design, which may restrict the generalizability of our findings. Furthermore, the two groups exhibited significant baseline differences (Table 1), including tumor location, histological type (all RT (+) patients had squamous cell carcinoma), and treatment indication. These structural imbalances are inherent reflection of current standard treatment protocols in Japan (i.e., definitive chemoradiotherapy is primarily used for squamous cell carcinoma, not adenocarcinoma). Due to the limited sample size and the high collinearity among these covariates, we were unable to perform robust multivariate analysis or matching procedures to adjust for these confounding factors. In addition, because most patients in the RT (+) group received approximately 50 Gy as part of standard definitive chemoradiotherapy, the distribution of radiation doses was highly imbalanced. Therefore, stratified analyses of radiation-related toxicities or dose-dependent effects were not feasible.

In clinical practice, careful monitoring of all adverse events remains essential when radiotherapy is combined with immune checkpoint inhibitor therapy.

## 6. Conclusions

In the present study, the combination of radiotherapy and ICI treatment did not affect the incidence or severity of irAEs in patients with esophageal and EGJ cancers. However, the mediastinum is included in the radiation field, and the effect on the immune system may be greater than in other cancers. ICIs also modulate the immune system. When combined, the incidence and severity of irAEs will be theoretically affected. ICI treatment combined with radiotherapy will play a great role in the treatment of patients with advanced esophageal and esophagogastric cancers. Treatment details and the profiles of irAEs should be carefully monitored, especially in patients who receive radiotherapy to the mediastinum, including the lymph nodes.

## Figures and Tables

**Table 1:** Baseline characteristics of the RT (+) and RT (-) groups.

		RT (+) (n = 35)	RT (-) (n = 23)	*p*-Value
Age, y (mean)		70.1	66.5	0.43
M:F		27:8	17:6	1.00
cStage	I–II/III–IV	5/30	3/20	0.74
Location	Thoracic/EGJ	35/0	15/8	1.00
Histology	SCC/AC	35/0	15/8	1.00
Indication for ICIs	Unresectable	14 (40%)	14 (61%)	<0.01
	Recurrence after surgery	1 (3%)	9 (39%)	
	Recurrence after radiotherapy	20 (57%)	0 (0%)	
ICI group	Group N	20 (57%)	15 (65%)	0.76
	Group P	10 (29%)	6 (26%)	
	Group NI	5 (14%)	2 (9%)	
RT dose	<30 Gy	3 (9%)		
	≥30 Gy <50 Gy	6 (17%)		
	≥50 Gy	26 (74%)		
RT field	Upper mediastinum	14 (40%)		
	Lower mediastinum	3 (9%)		
	Upper and lower mediastinum	18 (51%)		

SCC, squamous cell carcinoma; AC, adenocarcinoma; M, male; F, female; EGJ, esophagogastric junction; RT, radiotherapy; ICI, immune checkpoint inhibitor; N, nivolumab; P, pembrolizumab; NI, nivolumab plus ipilimumab.

**Table 2:** Profiles and grades of non-immune-related and immune-related adverse events in the RT (+) and RT (-) groups.

	RT (+)					RT (-)				
Grade	1	2	3	4	5	1	2	3	4	5
Leukopenia	3	7	4	0	0	3	1	4	0	0
Neutropenia	3	4	3	1	0	1	1	2	0	0
Anemia	19	9	1	0	0	9	9	2	0	0
Thrombocytopenia	7	1	0	0	0	4	1	2	0	0
Hepatic abnormalities	3	3	0	0	0	11	4	0	0	0
Renal abnormalities	11	2	0	0	0	2	6	0	0	0
irAE										
Adrenal abnormalities	1	1	3	0	0	1	3	1	0	0
Thyroid abnormalities	2	1	1	0	0	1	1	0	0	0
Pneumonitis	0	0	1	0	1	0	1	0	0	0
Dermatitis	1	1	0	0	0	0	1	0	0	0
Cytokine release syndrome	0	0	1	0	0	0	0	1	0	1
Intestinal abnormalities	0	0	0	0	0	0	1	1	0	0

RT, radiotherapy; irAE, immune-related adverse event.

**Table 3:** Baseline characteristics and grades of immune-related adverse events in the RT to ICI group and the ICI to RT group.

		RT to ICI (n = 21)	ICI to RT (n = 14)	*p*-Value
Age, y (average)		69.5	71.1	0.61
M:F		16:5	11:3	1.00
Stage	I–II/III–IV	5/16	0/14	0.07
irAE grade	0	16 (76%)	9 (64%)	0.73
	1	3 (14%)	1 (7%)	
	2	2 (10%)	1 (7%)	
	3	4 (19%)	2 (14%)	
	4	0 (0%)	0 (0%)	
	5	0 (0%)	1 (7%)	

M, male; F, female; RT, radiotherapy; ICI, immune checkpoint inhibitor; irAE, immune-related adverse event.

**Table 4:** Baseline characteristics and grades of immune-related adverse events in the short-interval and long-interval groups.

		Short-Term (n = 18)	Long-Term (n = 17)	*p*-Value
Age, y (average)		69.3	70.9	0.60
M:F		14:4	13:4	1.00
Stage	I–II/III–IV	0/18	5/12	0.02
irAE grade	0	12 (67%)	13(76%)	0.56
	1	3 (17%)	1 (6%)	
	2	1 (6%)	2 (12%)	
	3	2 (11%)	4 (24%)	
	4	0 (0%)	0 (0%)	
	5	1 (6%)	0 (0%)	

M, male; F, female; irAE, immune-related adverse event.

**Table 5:** Baseline characteristics and grades of immune-related adverse events in each ICI group.

		N (n = 35)	P (n = 16)	NI (n = 7)	*p*-Value
Age, y (average)		69.7	66.9	67.6	0.50
M:F		27:8	11:5	6:1	0.66
Stage	I–II/III–IV	4/31	2/16	2/5	0.48
	Thoracic/EGJ	29/6	15/1	6/1	0.58
	SCC/AC	28/7	15/1	7/0	0.22
Indication for ICI treatment	Unresectable	14 (40%)	14 (88%)	0 (0%)	0.01
	Recurrent after surgery	8 (23%)	0 (0%)	2 (29%)	
	Recurrent after RT	13 (37%)	2 (13%)	5 (71%)	
irAE grade	0	12 (67%)	13 (76%)	1 (14%)	0.01
	1	1 (6%)	0 (0%)	0 (0%)	
	2	3 (17%)	2 (12%)	4 (57%)	
	3	1 (6%)	2 (12%)	2 (29%)	
	4	0 (0%)	0 (0%)	0 (0%)	
	5	1 (6%)	0 (0%)	0 (0%)	

M, male; F, female; irAE, immune-related adverse event; ICI, immune checkpoint inhibitor; N, nivolumab; P, pembrolizumab; NI, nivolumab plus ipilimumab; SCC, squamous cell carcinoma; AC, adenocarcinoma; RT, radiotherapy.

## Data Availability

The data used and/or analyzed during the current study are available from the corresponding author upon reasonable request.

## Abbreviations

The following abbreviations are used in this manuscript:

ICI	Immune checkpoint inhibitor
irAE	Immune-related adverse event
RT	Radiotherapy
CTLA-4	Cytotoxic T lymphocyte-associated protein 4
PD-1	Programmed cell death 1
PD-L1	Programmed cell death ligand 1
EGJ	Esophagogastric junction
MHC	Major histocompatibility complex
UICC	Union for international cancer control
CTCAE	Common terminology criteria for adverse events
IMRT	Intensity-modulated radiation therapy
VMAT	Volumetric modulated arc therapy
CRS	Cytokine release syndrome
TME	Tumor microenvironment
ESMO	European society of medical oncology
NCCN	National comprehensive cancer network

## References

[1] Vanpouille-Box C., Formenti S.C., Demaria S. (2018). Toward Precision Radiotherapy for Use with Immune Checkpoint Blockers. Clin. Cancer Res..

[2] Nagasaki J., Ishino T., Togashi Y. (2022). Mechanisms of resistance to immune checkpoint inhibitors. Cancer Sci..

[3] Yoneda K., Kuwata T., Kanayama M., Mori M., Kawanami T., Yatera K., Ohguri T., Hisaoka M., Nakayama T., Tanaka F. (2019). Alteration in tumoral PD-L1 expression and stromal CD8-positive tumor-infiltrating lymphocytes after concurrent chemo-radiotherapy for non-small cell lung cancer. Br. J. Cancer.

[4] Charpentier M., Spada S., Van Nest S.J., Demaria S. (2022). Radiation therapy-induced remodeling of the tumor immune microenvironment. Semin. Cancer Biol..

[5] Deng L., Liang H., Burnette B., Beckett M., Darga T., Weichselbaum R.R., Fu Y.X. (2014). Irradiation and antPD-L1 treatment synergistically promote antitumor immunity in mice. Clin. Investig..

[6] Cheng X., Chen B., Wang S., Zhang J., Zhu J., Liu M., Liu S., Xi M. (2023). Association between lymphopenia and survival outcomes in esophageal carcinoma patients receiving combined immunotherapy and chemotherapy. Oncologist.

[7] Davuluri R., Jiang W., Fang P., Xu C., Komaki R., Gomez D.R., Welsh J., Cox J.D., Crane C.H., Hsu C.C. (2017). Lymphocyte nadir and esophageal cancer survival outcomes after chemoradiation therapy. Int. J. Radiat. Oncol. Biol. Phys..

[8] Brahmer J.R., Lacchetti C., Schneider B.J., Atkins M.B., Brassil K.J., Caterino J.M., Chau I., Ernstoff M.S., Gardner J.M., Ginex P. (2018). Management of immune-related adverse events in patients treated with immune checkpoint inhibitor therapy: American Society of Clinical Oncology Clinical Practice Guideline. J. Clin. Oncol..

[9] Haanen J., Carbonnel F., Robert C., Kerr K., Peters S., Larkin J., Jordan K. (2017). Management of toxicities from immunotherapy: ESMO Clinical Practice Guidelines for diagnosis, treatment and follow-up. Ann. Oncol..

[10] Anscher M.S., Arora S., Weinstock C., Amatya A., Bandaru P., Tang C., Girvin A.T., Fiero M.H., Tang S., Lubitz R. (2022). Association of radiation therapy with risk of adverse events in patients receiving immunotherapy: A pooled analysis of trials in the US Food and Drug Administration Database. JAMA Oncol..

[11] Postow M.A., Callahan M.K., Wolchok J.D. (2012). The antitumor immunity of ipilimumab: (T-cell) memories to last a lifetime?. Clin. Cancer Res..

[12] Sharma P., Hu-Lieskovan S., Wargo J.A., Ribas A. (2017). Primary, Adaptive, and Acquired Resistance to Cancer Immunotherapy. Cell.

[13] Dougan M., Luoma A.M., Dougan S.K., Wucherpfennig K.W. (2021). Understanding and treating the inflammatory adverse events of cancer immunotherapy. Cell.

[14] Lu C.S., Liu J.H. (2017). Pneumonitis in cancer patients receiving anti-PD-1 and radiotherapies: Three case reports. Medicine.

[15] Johnson D.B., Balko J.M., Compton M.L., Chalkias S., Gorham J., Xu Y., Hicks M., Puzanov I., Alexander M.R., Bloomer T.L. (2016). Fulminant myocarditis with combination immune checkpoint blockade. N. Engl. J. Med..

[16] Jaffray D.A., Gospodarowicz M.K., Gelband H., Jha P., Sankaranarayanan R., Horton S. (2015). Radiation therapy for cancer. Cancer: Disease Control Priorities.

[17] Postow M.A., Callahan M.K., Barker C.A., Yamada Y., Yuan J., Kitano S., Mu Z., Rasalan T., Adamow M., Ritter E. (2012). Immunologic correlates of the abscopal effect in a patient with melanoma. N. Engl. J. Med..

[18] Dovedi S.J., Adlard A.L., Lipowska-Bhalla G., McKenna C., Jones S., Cheadle EJStratford I.J., Poon E., Morrow M., Stewart R., Jones H. (2014). Acquired resistance to fractionated radiotherapy can be overcome by concurrent PD-L1 blockade. Cancer Res..

[19] Antonia S.J., Villegas A., Daniel D., Vicente D., Murakami S., Hui R., Yokoi T., Chiappori A., Lee K.H., de Wit M. (2017). Durvalumab after chemoradiotherapy in stage III non-small-cell lung cancer. N. Engl. J. Med..

[20] Hiniker S.M., Chen D.S., Reddy S., Chang D.T., Jones J.C., Mollick J.A., Swetter S.M., Knox S.J. (2012). A systemic complete response of metastatic melanoma to local radiation and immunotherapy. Transl. Oncol..

[21] Cooper J.S., Guo M.D., Herskovic A., Macdonald J.S., Martenson JAJr Al-Sarraf M., Byhardt R., Russell A.H., Beitler J.J., Spencer S., Asbell S.O. (1999). Chemoradiotherapy of locally advanced esophageal cancer: Long-term follow-up of a prospective randomized trial (RTOG 85-01). radiation therapy oncology group. JAMA.

[22] Lordick F., Mariette C., Haustermans K., Obermannová R., Arnold D. (2022). Oesophageal cancer: ESMO Clinical Practice Guidelines. Ann. Oncol..

[23] NCCN Clinical Practice Guidelines in Oncology: Esophageal and Esophagogastric Junction Cancers. Version 2023. https://www.nccn.org/.

[24] Shah M., Bennouna J., Doi T., Shen L., Kato K., Adenis A., Mamon H.J., Moehler M., Fu X., Cho B.C. (2021). KEYNOTE-975 study design: A Phase III study of definitive chemoradiotherapy plus pembrolizumab in patients with esophageal carcinoma. Future Oncol..

[25] Morisada M., Clavijo P., Moore E., Sun L., Chamberlin M., Van Waes C., Hodge J.W., Mitchell J.B., Friedman J., Allen C.T. (2017). PD-1 blockade reverses adaptive immune resistance induced by high-dose hypofractionated but not low-dose daily fractionated radiation. Oncoimmnology.

[26] Wu J., Deng R., Ni T., Zhong Q., Tand F., Li Y., Zhang Y. (2022). Efficacy and safety of radiotherapy/chemoradiotherapy combined with immune checkpoint inhibitors for locally advanced stages of esophageal cancer: A systematic review and meta-analysis. Front. Oncol..

[27] Zhang W., Yan C., Gao X., Li X., Cao F., Zhao G., Zhao J., Er P., Zhang T., Chen X. (2021). Safety and feasibility of radiotherapy plus camrelizumab for locally advanced esophageal squamous cell carcinoma. Oncologist.

